# Effect of Flaxseed Oil Cake Extract on the Microbial Quality, Texture and Shelf Life of Gluten-Free Bread

**DOI:** 10.3390/foods12030595

**Published:** 2023-01-31

**Authors:** Łukasz Łopusiewicz, Przemysław Łukasz Kowalczewski, Hanna Maria Baranowska, Łukasz Masewicz, Ryszard Amarowicz, Urszula Krupa-Kozak

**Affiliations:** 1 Center of Bioimmobilisation and Innovative Packaging Materials, Faculty of Food Sciences and Fisheries, West Pomeranian University of Technology, Klemensa Janickiego 35 Str., 71-270 Szczecin, Poland; 2 Department of Food Technology of Plant Origin, Poznań University of Life Sciences, 31 Wojska Polskiego Str., 60-624 Poznań, Poland; 3 Department of Physics and Biophysics, Faculty of Food Science and Nutrition, Poznań University of Life Sciences, 38/42 Wojska Polskiego Str., 60-637 Poznań, Poland; 4 Institute of Animal Reproduction and Food Research of Polish Academy of Sciences, Tuwima 10 Str., 10-748 Olsztyn, Poland

**Keywords:** flaxseed, gluten-free breadmaking, secoisolariciresinol diglucoside, microbial stability, LF NMR, texture profile, staling, by-product revalorization

## Abstract

Extending the shelf life of gluten-free bread (GFB) is a challenge. Mainly due to the ingredients used and their characteristics, GFB has numerous drawbacks such as unsatisfactory texture and rapid staling beyond a low nutritional value. In the present study, flaxseed oil cake extract (FOCE) was used to replace water (25–100%) in GFB formulations in order to test FOCE’s potential to reduce GFB staling and extend microbial stability. Texture (TPA test), water activity (LF NMR), acidity (pH measurements) and microbiological quality of GFBs were tested. Moreover, the content of a lignan with broad health-promoting potential, secoisolariciresinol diglucoside (SDG), in GFB with FOCE was analyzed. The results showed that the use of FOCE enriched experimental GFB in valuable SDG (217–525 µg/100 g DM) while not causing adverse microbiological changes. A moderate level (25–50%) of FOCE did not change the main texture parameters of GFB stored for 72 h, the quality of which was comparable to control bread without FOCE. Meanwhile, higher proportions of FOCE (75–100% of water replacement) shortened GFB shelf life as determined by water activity and texture profile, suggesting that GFB with FOCE should be consumed fresh. To summarize, FOCE at moderate levels can add value to GFBs without causing a drop in quality, while still fitting in with the idea of zero waste and the circular economy.

## 1. Introduction

Bakery products are consumed worldwide in large quantities and play an important role in human nutrition. Among bakery products, bread as one of the most common staple foods is eagerly consumed around the world every day [1,2]. However, for some consumers with gluten-related disorders (such as celiac disease, wheat allergy and non-celiac gluten sensitivity), consuming conventional wheat bread and other gluten-containing products is harmful. Currently, the only available treatment for gluten-related disorders is a strict and life-long gluten-free (GF) diet. Therefore, the demand for GF products continues to grow and the GF market has become one of the most important segments in the baking industry. GF breadmaking is a process that varies substantially from conventional breadmaking, mainly in the ingredients used and overall quality of the final product [3,4]. Many gluten-free breads (GFBs) available on the market have some disadvantages, such as: unattractive appearance (irregular crust and pale color), poor mouthfeel and flavor, unsatisfactory texture, low nutritional value and faster staling rate resulting in shorter shelf life when compared with conventional bread [3,4]. In addition, compared to conventional breads, they tend to have a much higher price and less availability. Another serious disadvantage that GFB is usually associated with is the inability to maintain the freshness of bread for an extended period of time. During storage, bread undergoes numerous physicochemical changes, ranging from hardening of the crumb, loss of crust crispness and organoleptic freshness, to a gradual decline in consumer acceptance. These changes are commonly referred to as staling, which is a complex but not well-understood process primarily associated with moisture redistribution, starch retrogradation, polymer reorganization and starch–protein interactions [3]. Over the last decade, considerable advances were made to improve technological, nutritional, sensory and functional characteristics of GFBs. The aforementioned challenges of GFBs are being addressed by testing new processing, fortification and storage techniques using a variety of ingredients that are sources of nutrients and bioactive compounds [5]. 

In this context, numerous studies have shown that plant-based by-products contain a substantial amount of nutrients, minerals, vitamins and phytochemicals, and thus can be considered as interesting additives to GFBs [3,6,7]. Among them, many phytochemicals are known for their important biological activities, such as antioxidant, anticancer and antimicrobial activity, and thus could play a role in the prevention and treatment of noncommunicable human diseases. For that reason, an increasing number of research focuses on the application of by-products in GFBs as low-cost sources of nutrients, bioactive compounds and functional ingredients [3,4]. Recent and on-going studies demonstrated that the use of by-products in the gluten-free food industry affords the possibility of improving the quality of many products [3,8]. 

Flaxseed (*Linum usitatissimum* L.), belonging to the Linaceae family, is one of the most important oilseed crops for industrial as well as for food purposes, and is making its mark in the world’s food supply as a functional food [9,10,11,12]. Numerous studies reported the production of high-quality, flaxseed-enriched cereal products with the desired health attributes exhibiting similar or improved shelf life compared to equivalent products [13,14]. Flaxseed seeds are commonly used as an ingredient in conventional breads for their high content of fiber and the omega-3 fatty acid—α-linolenic acid [9,10,15]. Flaxseed is also a rich source of an oligomeric complex containing the major lignan secoisolariciresinol diglucoside (SDG) [12,16]. SDG is a precursor plant lignan that is converted by bacteria in the colon to mammalian lignans known as enterodiol and enterolactone that are recognized to protect against hormone-dependent breast cancer and prostate gland cancer, and are also characterized by an anti-inflammatory activity, a laxative effect and alleviation of menopausal symptoms as well as osteoporosis [9,15,16]. The health benefits of flaxseed lignans are thought to be due to strong antioxidant activity, primarily as hydroxyl radicals scavengers, and also as an estrogenic and antiestrogenic compound due, in part, to the structural similarity to 17-β-estradiol [9]. In recent years, new perspectives for oilseed crops have revealed them to be a renewable and valuable source of by-products responding to the urgent need to transition towards a circular economy model based on the zero-waste concept [11,17]. After cold screw-pressed oil extraction from flaxseed seeds, a flaxseed oil cake (FOC) is obtained. This valuable and cheap by-product is underutilized in terms of food science and human food systems [18]. Only a few examples of FOC applications as an additive for foods are reported, for instance, in conventional bread [19] and sourdough bread [20]. However, FOC and FOC-derived extract (known as FOCE—flaxseed oil cake extract) were recently explored as raw materials for the development of plant-based dairy alternatives [21,22,23,24,25] and food additives [18,26,27,28], indicating their potential as a valuable by-product in food systems. FOCE consists of approximately 3% of dry matter, including: 14 mg/mL of proteins, 6.5 mg/mL of saccharides and 9.5 mg/mL of other extractable compounds (polyphenols, flavonoids, amino acids, mucilage, etc.) [23,25,26,27,28].

In fact, recently, Krupa-Kozak et al. [6] evaluated the impact of water replacement (25–100%) with FOCE on nutritional value, antioxidant properties and sensory quality of GFBs. This study indicated that GFBs with FOCE had an elevated nutritional and nutraceutical profile (up to 60% more proteins, significantly increased K, Mg and P levels). Moreover, the addition of FOCE favorably modified the technological parameters (increased specific volume, number of cells and height/width ratio, reduced density, average size and perimeter of cells), antioxidant potential and overall sensory quality of GFBs. However, the stability and staling process of experimental FOCE-enriched GFBs was not analyzed.

Therefore, this study aimed to investigate the potential of FOCE to reduce GFB staling and spoilage depending on its level in the formula in comparison with a control. The evaluation was performed using instrumental methods (TPA, LF NMR, pH measurements) and microbiological assays.

## 2. Materials and Methods

### 2.1. Composition of Experimental Gluten-Free Bread 

In the present research, a gluten-free bread (GFB) formulation with FOCE that was developed in our previous study was used [6]. Besides the main ingredients such as corn starch (HORTIMEX, Konin, Poland), potato starch (PPZ “Trzemeszno” Sp. Z o.o., Trzemeszno, Poland), pectin (RH-RS 150EEC; Herbstreith & Fox Jasło Sp. z o.o., Jasło, Poland), sugar, fresh yeast (Lesaffre Polska S.A., Wołczyn, Poland), rapeseed oil (ZT “Kruszwica” S.A., Kruszwica, Poland) and salt, the flaxseed oil cake extract (FOCE) was produced according to a previously described method [29] and used as a liquid ingredient replacing from 25 to 100% (*v*/*v*) of water in the control GFB formulation (Table 1). 

### 2.2. Preparation of Experimental Gluten-Free Bread

Starches and pectin were gently mixed for 5 min in a stainless steel bowl with a flat beater using a planetary mixer (KitchenAid Professional K45SS mixer; KitchenAid Europa, Inc, Brussels, Belgium). Salt, sugar and yeast were dissolved separately in water and then added to the dry mixture, together with oil. The batter was mixed for 12 min at speed 2. Batter samples (240 g) were placed in bread pans (10 cm × 10 cm × 9 cm, length, width and height, respectively) covered with baking paper and proofed for 40 min at 35 °C, 70% humidity. Breads were baked for 30 min at 220 °C in the laboratory oven (ZBPP, Bydgoszcz, Poland). Obtained bread loaves were cooled for 2 h at room temperature and then they were packed in clip-on plastic bags and kept in the dark for 24 h, 72 h and 120 h at room temperature (23 ± 1 °C) for further analysis. Products of two independent batches were analyzed. 

### 2.3. Analysis of Secoisolariciresinol Diglucoside 

#### 2.3.1. Extraction of SDG from the FOCE and GFB

A macromolecule of secoisolariciresinol diglucoside (SDG) was extracted from freeze-dried FOCE and experimental GFB according to the method developed by Johnsson et al. [30] with a slight modification. Briefly, the portions of FOCE (2 g) and GFBs (10 g) were extracted with a mixture (1:1; *v*/*v*) of 1,4-dioxane and 95% ethanol (40 mL and 100 mL, respectively) in test tubes by shaking for 16 h in a 60 °C water bath. After 20 min centrifugation at 5000× *g*, washing, re-centrifugation and in vacuo evaporation of the liquid phase at 40 °C, the extracts were subjected to alkaline hydrolysis for 2 days under constant rotation using 2 M aqueous sodium hydroxide. After hydrolysis, the samples were acidified to pH 3 using 2 M sulfuric acid and their volume was adjusted to 25 mL in volumetric flasks.

#### 2.3.2. High-Performance Liquid Chromatography (HPLC) Analysis of SDG

For the HPLC analysis of SDG, a Shimadzu system (Shimadzu Corp., Kyoto, Japan) consisting of two LC-10AD pumps, an SCTL 10 A system controller, an SPD-M 10 A photo-diode array detector and a prepacked LUNA C 18 (4 × 259 mm, 5 μm, Phenomenex) was used. A flow rate of 1 mL/min and gradient elution of acetonitrile-water-acetic acid (5:93:2, *v*/*v*/*v*) [solvent A] and of acetonitrile-water-acetic acid (40:58:2, *v*/*v*/*v*) [solvent B], 0–50 min solvent B from 0 to 100%, were used [31]. The concentration of the sample dissolved in methanol was 2 mg/mL; the injection volume was 20 μL; the separation of compounds was monitored at 280 nm. The SDG peaks were identified and quantified by comparison with the SDG standard. A linear HPLC calibration curve for standard SDG was obtained for the concentration range of 0, 20, 40, 80, 120 and 160 µg/mL (R value 0.997). The repeatability of the whole method was tested by simultaneous analysis of 6 replicates and the coefficient of variation was <5%. The SDG standard was obtained from flaxseed alkaline hydrolysate using semi-preparative HPLC (LUNA C 18 (10 × 250 mm, 5 μm, Phenomenex)).

### 2.4. Characteristics of Experimental GFB with FOCE

#### 2.4.1. Microbiological Analysis of Samples and pH Measurements

The representative sample cuts (crumb with crust—10 g) were collected and aseptically introduced into a sterile stomacher bag, then diluted with 90 mL of sterile physiological saline (0.9%). The samples were homogenized in a Bag Mixer (Interscience, Saint-Nom-la-Brèteche, France) for one minute and appropriate decimal dilutions were prepared in sterile buffered peptone water (Merck, Darmstad, Germany). Total mesophilic microbial counts were enumerated on Plate Count Agar (Merck, Darmstad Germany), whereas coliforms were determined on Violet Red Bile Glucose Agar (Merck, Darmstad, Germany), both after incubation at 37 °C under aerobic conditions for 48 h. The presence of *Bacillus* sp. was determined on Mannitol Yolk Polymyxin B Agar (Merck, Darmstad, Germany). Fungal counts were determined on Sabouraud Agar amended with chloramphenicol (0.005%) (Merck, Darmstad, Germany) at 25 °C for 72 h under aerobic conditions. The enumeration of microorganisms was performed in triplicate (by counting plates with 30–300 colonies) and the viable cell counts were expressed as CFU/g of the samples.

A pH meter (CP-411, Elmetron, Zabrze, Poland) was used to determine the pH of the samples by immersing the device’s probe (at 25 °C) directly into the samples homogenized in saline.

#### 2.4.2. Moisture Content and Instrumental Evaluation of Texture Profile

The moisture content of the crumb of experimental GFBs was determined in fresh (24 h after baking) and stored (72 h and 120 h) GFBs according to the standard method (AOAC method 926.05) [32]. 

The texture profile (TPA test) of experimental GFBs was analyzed 24 h, 72 h and 120 h after baking using a TA.HDplus Texture Analyser (Stable Micro Systems Ltd., Godalming, UK) equipped with a 30-kg load. The 25 mm middle slice of GFBs underwent a double compression cycle of up to 40% deformation of its original height with a 35 mm flat-end aluminum compression disc (probe P/35) to give a two-bite texture profile curve [33]. The selected settings were as follows: pre-test/test/post-test speed, 2.0 mm/s; relaxation time, 5 s; force,10 g; and trigger, mode auto. The following textural parameters were determined: hardness, springiness, cohesiveness, gumminess, chewiness and resilience, as calculated by the software of the texturometer. Six replicates were analyzed for each kind of GFB.

#### 2.4.3. Low-Field NMR Relaxometry

LF NMR relaxation times were analyzed using a pulse NMR spectrometer MSL30 operating at 20 MHz (WL Electronics, Poznań, Poland) according to the method described in detail by Kowalczewski et al. [34]. The inversion–recovery (180−t−90) pulse sequence was applied for measurements of the spin–lattice (T_1_) relaxation times, while the measurements of the spin–spin (T_2_) relaxation times were taken using the pulse train of the Carr–Purcell–Meiboom–Gill spin echoes [35].

#### 2.4.4. Water Activity

Measurements of water activity were performed independently on each of the 5 days of measurement using an analyzer of water diffusion and activity ADA-7 (COBRABID, Poznań, Poland) with a system of automatic time recordings of the water evacuation runs from individual samples. Each time, samples of bread crumb (Ø = 15 mm) were cut from new loaves and stored at room temperature (22 °C). The process was carried out for six repetitions (n = 6) each day. Before the measurement, the chamber was dried to a water activity of 0.10. The temperature was stabilized at 20.0 ± 0.1 °C using Peltier modules. The duration of one measurement was set at 1200 s. The measured water activity was used to calculate the water transport according to the phenomenological model described previously by Masewicz et al. [36]:

$$a_{w}\left(t\right)=a_{r}+\left(a_{0}-a_{p}\right)e^{-V_{D}t}+\left(a_{p}-a_{r}\right)e^{-V_{p}t}$$

where: a_w_(t)—temporary water activity value, a_0_—initial water activity, a_p_—limit water activity (intermediate), a_r_—water activity at equilibrium condition (final), V_D_—transport rate, V_p_—rate of the surface conduction.

### 2.5. Statistical Analysis

The data reported in tables and figures are presented as the average values and standard deviations of triplicate observations unless otherwise stated. The differences between experimental GFBs were analyzed by one-way analysis of variance (ANOVA) with Tukey’s multiple comparison test (*p* < 0.05) using GraphPad Prism (version 8.0.0 for Windows, GraphPad Software, San Diego, CA, USA).

## 3. Results and Discussion

### 3.1. Identification and Content of Secoisolariciresinol Diglucoside (SDG) in FOCE and Experimental GFB with FOCE 

**Figure 1:**
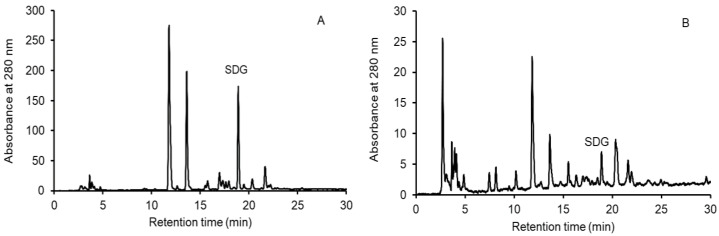
HPLC chromatograms showing SDG (t R 18.921) along with other components: (**A**) chromatogram of the hydrolyzed FOCE; (**B**) chromatogram of the hydrolyzed FOCE100%.

The SDG of the hydrolyzed extract of oligomers extracted from freeze-dried FOCE and experimental GFBs with FOCE is shown along with other components in the example HPLC chromatograms (Figure 1). As can be seen in Figure 1A, the SDG of FOCE eluted at between 18 and 20 min and showed a relatively high peak. This distinctive peak was also detected in chromatograms of GFB with FOCE (Figure 1B). The SDG peaks were identified by comparison with the SDG standard.

The content of SDG in FOCE quantified based on the standard was 0.261 ± 0.018 g/100 g DM. As expected, the amount of SDG in the experimental GFBs depended on the level of water exchange with FOCE and ranged from 217 to 526 µg/100 g DM (Table 2). SDG level was lower than that reported by Strandas et al. [15] for conventional bread containing whole flaxseed seeds. Whole flaxseed seeds are reported to contain from 0.9 to 3 g/100 g DM of SDG and in fact are the most abundant source of SDG [9]. However, it should be emphasized that technological processing such as FOCE production can affect lignan content at any stage of the process. The amount of SDG increased with the level of FOCE in the GFB formula, therefore bread in which water was replaced with FOCE could be considered a source of valuable SDG in a gluten-free diet.

### 3.2. Characteristics of Experimental GFB with FOCE

#### 3.2.1. Microbial Quality and Acidity Changes of GFB with FOCE during Storage

Overall, significantly lower levels of microorganisms were found in GFBs than recommended by norms for conventional breads, indicating the purity of the production process (Table 3). However, it should be emphasized that the breads were not produced and stored under strictly sterile conditions, hence a certain level of microorganisms was found. Particularly noteworthy is the total absence of coliforms and *Bacillus* sp., which indicates the quality of the ingredients used. Although FOCE can be considered as a factor that introduces additional nutrients to microorganisms, there were no statistically significant differences between control and GFBs with FOCE after 24 h and 72 h of storage (*p* > 0.05). After 120 h of storage, a significant difference in fungal counts was observed between control samples and GFBs with FOCE (*p* < 0.05). In addition to reports of fungistatic activity of flax extracts, it is also reported that this activity is poorly preserved after heat treatment [37,38]. It seems, therefore, that the increased presence of fungal counts in the control bread may be partly attributed to increased acidity, which to some extent favors mold growth. As can be seen, the presence of FOCE, which, due to its protein and polysaccharide content, may have buffering properties to some extent, significantly influenced the lower increase in acidity of GFBs after 120 h of storage (pH 4.04 ± 0.02 and 4.22 ± 0.11, for control bread and bread with 100% of FOCE, respectively).

Bread is a perishable product and adverse changes begin to appear in it immediately after baking [5]. These processes are related both to partial loss of moisture, i.e., bread staling (without the anticipation of microorganisms), as well as to the development of bacteria, molds and yeast. Flour as the basic ingredient in conventional bread production is often contaminated with microorganisms found on cereal grains [39]. Its colonization by microorganisms, in addition to the contamination of the grain, is also influenced by the microbiological state of the milling equipment, packaging and the premises where the raw material is stored. Molds found in flour are mostly represented by the genera *Aspergillus*, *Penicillium* and *Fusarium*, as well as species of the genera *Cladosporium* and *Alternaria*. Their development can occur at flour moisture content above 15% and the result is a change in organoleptic characteristics, an increase in acidity and a loss of baking properties caused by deterioration of gluten quality. In addition to molds, a diverse microflora can be found in flour. There may be coliforms and representatives of the *Achromobacter* sp., *Flavobacterium* sp., *Sarcina* sp., *Micrococcus* sp., *Alcaligenes* sp. and *Serratia* sp. However, the most common is the presence of *Bacillus* sp. and their spore forms. It should be noted that most GFBs, which are often based on pure starches, require an extra amount of water, thus are more prone to stale than their gluten-containing counterparts [3]. Thus, their exposure to microbial contamination, due to the lack of use of flour, may be mainly due to the hygienic conditions of the production process and storage. However, it is well known that high water content facilitates microbial growth. Maintaining a good microbiological quality of bread involves the use of flour free of microorganism contamination, consistent with the recipe, preparation of the dough, proper conduct of the process of baking and proper storage conditions. Commercial preservatives can extend the shelf life of food products, but they can adversely affect the sensory properties of food and are not seen as “green label” ingredients, so it was decided not to add preservatives in the GFB formulation. 

#### 3.2.2. Moisture and Texture Profile of GFB with FOCE during Storage

The level of FOCE in the GFB formula did not affect the moisture of the fresh crumb (24 h after baking), as determined previously [6], and therefore, as expected, no significant differences were detected in the crumb moisture of the GFBs stored for 120 h (Supplementary Materials Figure S1). The moisture of FOCE-containing GFBs ranged from 53.4 to 55% and was comparable to the control (53.4%). However, assessing the impact of the 120 h storage, a significant (*p* < 0.05) reduction in moisture was detected in all GFBs in comparison to their fresh counterparts. The highest moisture loss was determined in FOCE50% (14.7%), followed by FOCE75% (12.6%) and FOCE100% (8.5%). In the case of the control, the moisture of the crumb decreased by 7.5% after the 120-hour storage. The observed reduction of moisture in bread crumbs resulting from storage is a characteristic phenomenon that occurs due to a migration of moisture from the inner bread part (crumb) toward its surface (crust) [40].

**Figure 2:**
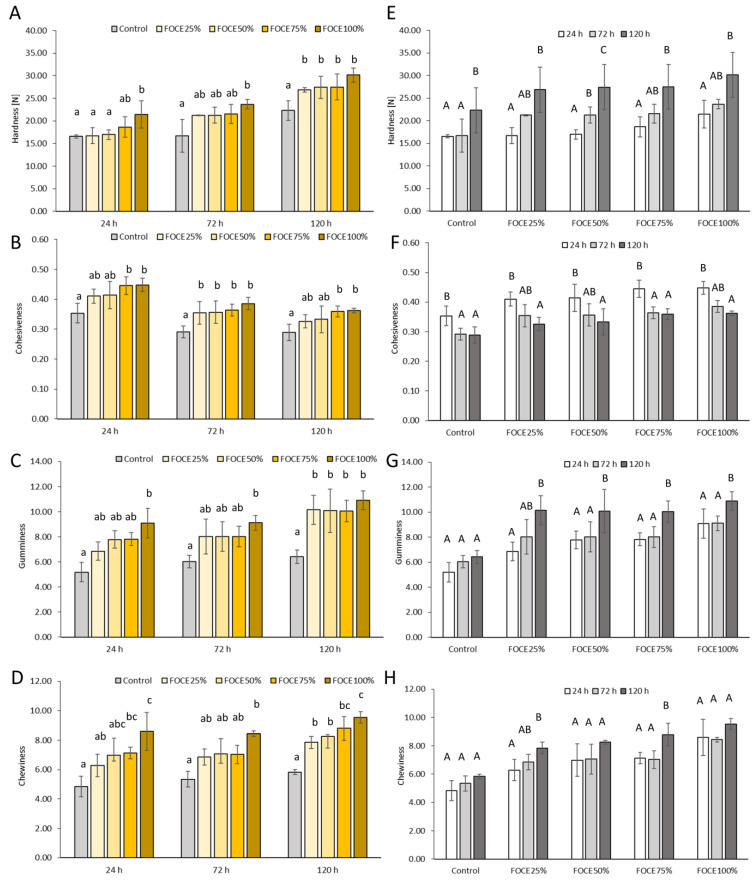
Changes in the hardness, cohesiveness, gumminess and chewiness of experimental GFBs related to FOCE level (**A**–**D**) and storage time (**E**–**H**). ^a–c^ different lowercase letters indicate significant differences between GFBs with different FOCE levels at the same storage time (*p* < 0.05); ^A–C^ different capital letters indicate significant differences between storage times for the same bread (*p* < 0.05).

Changes in the texture profile related to FOCE level and storage time are presented in Figure 2. The instrumental texture analysis was carried out at three time intervals (24 h, 72 h and 120 h after baking) by subjecting the middle slice of the experimental GFBs to a double compression cycle to determine hardness, elasticity, cohesiveness, gumminess, chewiness and resilience. Analyzing the hardness of the fresh GFBs, it was detected that samples with low- to moderate-FOCE levels (25–75%) were soft like the control GFB (Figure 2A). Similarly, the replacement of up to half of the water with FOCE in the GFB formula did not change the cohesiveness, gumminess and chewiness of fresh crumbs (Figure 2B–D), which were comparable to the control. However, a fresh GFB with the highest percentage of FOCE behaved differently. In particular, FOCE100%, in which all the water was replaced with the extract of flaxseed oil cake, became significantly (*p* < 0.05) harder (Figure 2A), more cohesive (Figure 2B) and gummy (Figure 2C), and required longer chewing (Figure 2D) before swallowing than the control. Crumb texture is influenced by many variables, including moisture, proteins and dietary fiber content, baking conditions and loaf volume [41]. Laboratory-produced FOCE is extracted from FOC, which is a flaxseed oil post-production waste. It is a liquid medium rich in nutritional macronutrients that have unique characteristics due to the presence of proteins and gums with synergistic water-holding and oil-binding abilities [29] that make FOCE a promising food stabilizer [18]. However, the loss of softness and deterioration of textural quality detected in FOCE100% could arise from the excessive concentration of mucilage gums and dietary fiber (cellulose, lignin) that may negatively affect the textural attributes of bread [42]. Texture plays a crucial role in the consumers’ perception of bread quality [43]. TPA results obtained in the present study correspond to some extent with the results of our previous sensory qualitative descriptive analysis (QDA) [6], where texture characteristics of GFBs were assessed by qualified panelists. According to QDA, GFBs containing FOCE did not differ meaningfully from the control in terms of chewiness and adhesiveness, while they were more elastic than the control when examined manually by finger pressing. Although differences between the instrumental and sensorial methods of texture analysis resulting from the variability caused by the psychological and physiological variability of human sensory reactions exist [44], both TPA and QDA support the conclusion that a moderate amount of FOCE rather than a complete replacement of water in the recipe is more suitable for obtaining a soft and elastic GFB.

No differences have been detected in hardness, gumminess and chewiness between the control and GFBs containing FOCE ranging from 25 to 75% stored for 72 h (Figure 2A,C,D), although the crumb cohesiveness increased significantly in all FOCE-containing breads (Figure 2B), irrespectively of its percentage. Nevertheless, all GFBs with FOCE were significantly (*p* < 0.05) harder, gummier and chewier than the control crumb after prolonged 120 h storage (Figure 2A,C,D), regardless of the FOCE level. This could be influenced by the reduction of crumb moisture (Supplementary Materials Figure S1), which, similarly to starch retrogradation, is involved in crumb hardening and bread staling [40,45]. 

Evaluating the storage time-related changes in the texture profile of the experimental GFBs with FOCE, it was shown that in general, 72-hour storage had no impact on the majority of the textural parameters. All examined crumbs of GFBs, regardless of the FOCE level, were as soft as their fresh counterparts (Figure 2E), except for FOCE50%, which was significantly (*p* < 0.05) harder than its fresh counterpart. Additionally, no significant changes in the crumbs’ gumminess (Figure 2G) and chewiness (Figure 2H) were observed after 72-hour storage. On the other hand, the cohesiveness of the control was significantly reduced, while such texture deterioration was not observed in the majority of GFBs with FOCE (Figure 2F). Further storage (120 h) resulted in crumb hardening and affected negatively the remaining textural parameters of all experimental GFBs. Springiness and resilience were not affected by the level of FOCE in the formula nor by the storage time (Supplementary Materials Table S1).

#### 3.2.3. Changes in Water Behavior of GFBs with FOCE during Storage

The changes observed macroscopically in texture analysis (described in Section 3.2.1) are related to the changes observed at the molecular level. During storage, the main ingredients of gluten-free bread, i.e., starch and water, change significantly. Literature data broadly describe the influence of starch transformations in the staling process [46], but changes in water dynamics also play an important role during the storage of bread. To observe progressive changes during GFB storage, water behavior analyses were performed daily (from 1st to 5th day after baking) using LF NMR. This is a noninvasive and nondestructive method applied in the analysis of the proton relaxation phenomena occurring as a result of electromagnetic field energy adsorption in various biological samples that is especially useful in food analysis [47,48,49,50]. LF NMR enables the study of differences in the molecular mobility of various food ingredients and the results are reflected in the longitudinal (T1) and transverse (T2) relaxation times of protons, mostly from water or fats. The spin–lattice (T1) relaxation time describes the transfer of previously absorbed energy from the spin to the surrounding environment, while the spin–spin (T2) relaxation time describes the transfer of previously absorbed energy from the spin to neighboring spins. The first is related to the ratio of free to entrapped water in the tertiary structure of biopolymers and the second is related to the dynamics of water molecules [47,49,50]. The results of measurements of spin–lattice (T_1_) and spin–spin (T_2_) relaxation times in the FOCE-containing GFBs are presented in Table 4. Two components of the spin–spin T_2_ relaxation time (T_21_ and T_22_) were observed, which is consistent with the results of the analysis of crumbs of gluten-free and wheat bread [51].

Measurements of relaxation times indicate that the replacement of water with FOCE reduces the amount of bulk water to bound water fraction. This is manifested by a decrease in the value of spin–lattice T_1_ relaxation times (Table 4) and may be explained by the fact that FOCE contains compounds with high water binding capacity, which was confirmed by the previous study indicating the presence of proteins and gums in FOCE [29]. Storage studies have shown an increase in the value of relaxation times between the second and the third day of storage, which is related to the release of water particles from the water complexes with biopolymers formed during the mixing of the dough and baking. During the following days of storage, a monotonic decrease in the values of the relaxation times was observed. The starch retrogradation process manifests itself in the formation of a secondary crystal structure [52,53,54], hence the observed decrease in the values of T_1_, T_21_ and T_22_. A comparison of the time changes of relaxation times for individual GFBs with FOCE shows that FOCE significantly influences the dynamics of molecules of the bound water fraction. The values of the T_21_ relaxation times are smaller the greater the replacement of water by FOCE is. After 4 days of storage, all analyzed GFBs have similar T_22_ values, so the use of FOCE in the recipe after this storage time no longer determines the possibility of molecular movements of water in this fraction. Importantly, changes in spin–lattice T_1_ relaxation times in the control and FOCE25% follow a similar course, with the minimum observed on the third day of storage (Table 4). The addition of FOCE above 25% causes a monotonic decrease in the T_1_ value after the third day of storage, which can be interpreted as an acceleration of water evacuation from the crumb. This is also confirmed by the time-dependent changes in the long component of the spin–spin relaxation time (T_22_). The decrease in T_22_ values can be explained by the starch retrogradation process [55] and/or water redistribution in the amorphous regions of crumbs [56,57].

**Figure 3:**
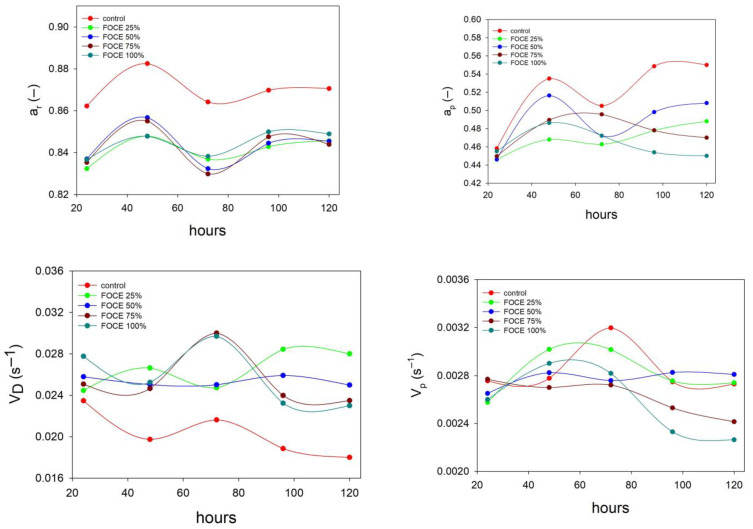
The results of measurements of the water activity of FOCE-containing GFBs.

The water content, but most of all the water activity, plays an important role in the formation of the structure and staling of the bread [58], therefore the analysis of changes in the equilibrium value of water activity (a_r_) and the transport rate of water (V_D_) in the crumb during the storage of bread was carried out. Changes in the values of both parameters in FOCE-containing GFBs compared to control confirm the significant influence of FOCE on water activity (Figure 3). Both parameters, like the values of relaxation times (Table 4), reach their maximum during 2–3 days of storage and then decrease monotonically. It was noted that during this storage time, the values of the intermediate water activity (a_p_) showed the greatest changes. It can therefore be concluded that this is the key shelf life time of bread, when the most important changes in water binding take place due to conformational changes in biopolymers [59,60]. Therefore, it can be concluded that the greater the share of FOCE in the bread recipe, the faster the staling process after 2–3 days. In the case of FOCE25%, the durability of the bread does not differ from the control.

## 4. Conclusions

Flaxseed oil cake extract is a valuable product obtained from a by-product of the oil industry (flaxseed oil cake), the use of which in the recipe of experimental bread allowed it to be enriched with the bioactive lignan SDG, which has health-promoting properties. However, those who will benefit from the supply of SDGs are presumably only those who have a gut microbiota capable of converting this compound into enterolactone and enterodiol, but this supposition requires further research with consumer involvement. Based on the results obtained, it can be concluded that a moderate FOCE level (25–50%) did not change the main texture parameters of GFB stored for 72 h, whose quality was comparable to the control bread without FOCE. However, a higher water replacement with FOCE (75–100%) shortened GFB shelf life as determined based on the water activity and texture profile. FOCE, due to its composition (proteins and polysaccharides content), may cause a faster loss of freshness, but it should be emphasized that the addition of FOCE could enrich GFB consumers’ with a valuable functional component with health-promoting activity (lignan SDG). In conclusion, the application of FOCE at a moderate level adds value to GFBs without causing a drop in its quality, while still fitting in with the idea of zero waste and the circular economy.

## Figures and Tables

**Table 1:** Ingredients of experimental gluten-free bread with FOCE.

Ingredient (%)	Control	FOCE25%	FOCE50%	FOCE75%	FOCE100%
Corn starch	36.7	36.7	36.7	36.7	36.7
Potato starch	8.9	8.9	8.9	8.9	8.9
Pectin	2.2	2.2	2.2	2.2	2.2
Sugar	2.8	2.8	2.8	2.8	2.8
Salt	0.8	0.8	0.8	0.8	0.8
Oil	1.4	1.4	1.4	1.4	1.4
Fresh yeast	2.8	2.8	2.8	2.8	2.8
FOCE ^1^	-	11.1	22.2	33.3	44.4
Water	44.4	33.3	22.2	11.1	-

^1^ FOCE—Flaxseed oil cake extract.

**Table 2:** Amount of SDG in the experimental GFBs with FOCE.

	Control	FOCE25%	FOCE50%	FOCE75%	FOCE100%
SDG (µg/100 g DM)	Nd	217 ± 11 ^a^	287 ± 14 ^b^	345 ± 10 ^c^	526 ± 33 ^d^

Nd—not detected. Means with different lowercase letters are significantly different at *p* < 0.05.

**Table 3:** Microbial counts and acidity of GFBs during storage time.

	Total Mesophilic Bacteria [CFU/g]	Fungi [CFU/g]	Coliforms [CFU/g]	*Bacillus* sp. [CFU/g]	pH
24 h					
Control	<10	<10	Nd	Nd	4.21 ± 0.11 ^a^
FOCE25%	<10	<10	Nd	Nd	4.22 ± 0.24 ^a^
FOCE50%	<10	<10	Nd	Nd	4.23 ± 0.08 ^a^
FOCE75%	<10	<10	Nd	Nd	4.26 ± 0.18 ^a^
FOCE100%	<10	<10	Nd	Nd	4.27 ± 0.10 ^b^
72 h					
Control	1.81 × 10^1^ ± 0.09 ^a^	1.78 × 10^1^ ± 0.12 ^a^	Nd	Nd	4.13 ± 0.05 ^a^
FOCE25%	1.67 × 10^1^ ± 0.23 ^b^	1.54 × 10^1^ ± 0.52 ^b^	Nd	Nd	4.15 ± 0.14 ^a^
FOCE50%	1.45 × 10^1^ ± 0.54 ^c^	0.78 × 10^1^ ± 0.57 ^c^	Nd	Nd	4.17 ± 0.12 ^a^
FOCE75%	1.99 × 10^1^ ± 0.23 ^d^	1.22 × 10^1^ ± 0.13 ^d^	Nd	Nd	4.19 ± 0.14 ^a^
FOCE100%	1.44 × 10^1^ ± 0.19 ^c^	1.09 × 10^1^ ± 0.85 ^e^	Nd	Nd	4.28 ± 0.07 ^b^
120 h					
Control	2.03 × 10^1^ ± 0.43 ^a^	5.33 × 10^3^ ± 0.12 ^a^	Nd	Nd	4.04 ± 0.02 ^a^
FOCE25%	1.88 × 10^1^ ± 0.43 ^a^	2.08 × 10^2^ ± 0.65 ^b^	Nd	Nd	4.09 ± 0.08 ^a^
FOCE50%	1.55 × 10^1^ ± 0.57 ^b^	1.39 × 10^2^ ± 0.25 ^c^	Nd	Nd	4.16 ± 0.22 ^a^
FOCE75%	2.12 × 10^1^ ± 0.69 ^c^	1.98 × 10^2^ ± 0.35 ^d^	Nd	Nd	4.18 ± 0.28 ^a^
FOCE100%	1.28 × 10^1^ ± 0.08 ^d^	1.88 × 10^2^ ± 0.87 ^e^	Nd	Nd	4.22 ± 0.11 ^b^

Values are means ± standard deviation of triplicate determinations. Means with different lowercase letters in the same column at specific time intervals (24 h, 72 h, 120 h) are significantly different at *p* < 0.05. Nd—not detected.

**Table 4:** LF NMR relaxometry measurement results of FOCE-containing GFBs.

	T_1_ (ms)	T_21_ (ms)	T_22_ (ms)
24 h			
Control	151.46 ± 0.85	0.81 ± 0.19	3.49 ± 0.10
FOCE25%	136.05 ± 2.20	0.74 ± 0.15	3.14 ± 0.08
FOCE50%	139.00 ± 2.23	0.53 ± 0.13	3.09 ± 0.03
FOCE75%	137.86 ± 2.20	0.44 ± 0.11	3.33 ± 0.11
FOCE100%	139.70 ± 1.46	0.44 ± 0.07	3.47 ± 0.05
48 h			
Control	153.39 ± 1.27	1.76 ± 0.35	4.19 ± 0.54
FOCE25%	141.73 ± 1.28	1.34 ± 0.26	3.67 ± 0.99
FOCE50%	144.06 ± 1.14	1.08 ± 0.13	3.39 ± 0.08
FOCE75%	143.12 ± 0.79	0.95 ± 0.05	3.64 ± 0.69
FOCE100%	141.18 ± 1.30	0.81 ± 0.08	3.68 ± 0.09
72 h			
Control	151.87 ± 1.11	1.69 ± 0.56	3.87 ± 0.59
FOCE25%	141.00 ± 1.65	1.41 ± 0.38	3.39 ± 0.40
FOCE50%	142.85 ± 1.27	1.17 ± 0.53	3.31 ± 0.94
FOCE75%	142.35 ± 0.78	1.01 ± 0.37	2.97 ± 0.62
FOCE100%	139.86 ± 0.75	0.64 ± 0.11	3.34 ± 0.06
96 h			
Control	152.20 ± 0.91	0.92 ± 0.09	3.58 ± 0.10
FOCE25%	142.15 ± 1.37	0.60 ± 0.11	2.98 ± 0.07
FOCE50%	139.99± 0.89	0.64 ± 0.09	3.18 ± 0.94
FOCE75%	136.84± 1.71	0.68 ± 0.06	2.84 ± 0.05
FOCE100%	137.59 ± 1.13	0.57 ± 0.05	3.28 ± 0.06
120 h			
Control	153.66 ± 1.36	0.80 ± 0.08	3.40 ± 1.03
FOCE25%	142.88 ± 1.45	0.54 ± 0.12	2.80 ± 0.70
FOCE50%	138.17 ± 0.99	0.60 ± 0.04	3.00± 0.70
FOCE75%	135.12 ± 2.20	0.60 ± 0.08	2.78 ± 0.80
FOCE100%	137.00 ± 0.87	0.52 ± 0.09	3.20 ± 0.10

## Data Availability

Data are contained within the article.

## Supplementary Materials

The following supporting information can be downloaded at: https://www.mdpi.com/article/10.3390/foods12030595/s1, Figure S1: Changes in the moisture of experimental GFBs related to FOCE level (A) and storage time (B); Table S1: Instrumental texture profile of experimental GFBs with FOCE during storage.
