# Supplementary material for: Effect of Flaxseed Oil Cake Extract on the Microbial Quality, Texture and Shelf Life of Gluten-Free Bread
Source: Foods. 2023 Jan 31;12(3):595. doi: 10.3390/foods12030595 (PMC9914225; doi:10.3390/foods12030595)
Supplement: Supplementary file 1 [file foods-12-00595-s001.zip › foods-2170541-supplementary.pdf]

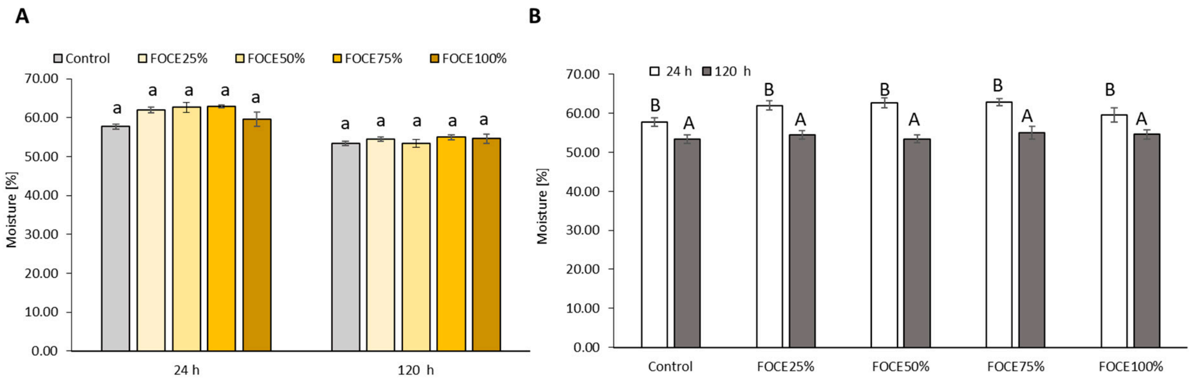

Supplementary Materials Figure S1. Changes in the moisture of experimental GFBs related to FOCE level (A) and storage time (B). ^{a} similar lowercase letters indicate no significant differences between GFBs with different FOCE levels at the same storage time ($p < 0.05$); $^{A-B}$ different capital letters indicate significant differences between storage times for the same bread ($p < 0.05$).

Suppl. Material. Table S1. Instrumental texture profile of experimental GFBs with FOCE during storage.

	Hardness [N]	Springiness	Cohesiveness	Gumminess	Chewiness	Resilience
			24 h			
Control	$16.54^{aA} \pm 0.37$	$0.90^{aA} \pm 0.03$	$0.35^{aB} \pm 0.03$	$5.18^{aA} \pm 0.78$	$4.84^{aA} \pm 0.70$	$0.15^{aA} \pm 0.03$
FOCE25%	$16.72^{aA} \pm 1.77$	$0.92^{aA} \pm 0.02$	$0.41^{abB} \pm 0.02$	$6.85^{abA} \pm 0.73$	$6.28^{abA} \pm 0.75$	$0.17^{aA} \pm 0.01$
FOCE50%	$16.96^{aA} \pm 1.04$	$0.90^{aA} \pm 0.04$	$0.41^{abB} \pm 0.05$	$7.79^{abA} \pm 0.71$	$6.99^{abcA} \pm 1.14$	$0.19^{aA} \pm 0.02$
FOCE75%	$18.65^{abA} \pm 2.22$	$0.92^{aA} \pm 0.02$	$0.45^{bB} \pm 0.03$	$7.82^{abA} \pm 0.52$	$7.13^{bcA} \pm 0.41$	$0.20^{aA} \pm 0.03$
FOCE100%	$21.43^{bA} \pm 3.06$	$0.91^{aA} \pm 0.03$	$0.45^{bB} \pm 0.02$	$9.08^{bA} \pm 1.18$	$8.60^{cA} \pm 1.27$	$0.20^{aA} \pm 0.02$
			72 h			
Control	$16.69^{aA} \pm 3.64$	$0.89^{aA} \pm 0.02$	$0.29^{aA} \pm 0.02$	$6.04^{aA} \pm 0.50$	$5.35^{aA} \pm 0.54$	$0.12^{aA} \pm 0.01$
FOCE25%	$21.22^{aAB} \pm 0.12$	$0.91^{aA} \pm 0.03$	$0.35^{bAB} \pm 0.04$	$8.01^{abAB} \pm 1.38$	$6.86^{abAB} \pm 0.56$	$0.14^{aA} \pm 0.03$
FOCE50%	$21.27^{aB} \pm 1.77$	$0.89^{aA} \pm 0.03$	$0.36^{bAB} \pm 0.04$	$8.02^{abA} \pm 1.19$	$7.06^{abA} \pm 1.06$	$0.15^{aA} \pm 0.03$
FOCE75%	$21.55^{aAB} \pm 2.11$	$0.88^{aA} \pm 0.04$	$0.36^{bA} \pm 0.02$	$8.02^{abA} \pm 0.83$	$7.04^{abA} \pm 0.63$	$0.15^{aA} \pm 0.01$
FOCE100%	$23.67^{bAB} \pm 1.03$	$0.89^{aA} \pm 0.04$	$0.39^{bAB} \pm 0.02$	$9.11^{bA} \pm 0.59$	$8.44^{bA} \pm 0.17$	$0.15^{aA} \pm 0.01$
			120 h			
Control	$22.31^{aB} \pm 2.15$	$0.91^{aA} \pm 0.05$	$0.29^{aA} \pm 0.03$	$6.41^{aA} \pm 0.52$	$5.84^{aA} \pm 0.17$	$0.12^{aA} \pm 0.01$
FOCE25%	$26.88^{bC} \pm 0.48$	$0.92^{aA} \pm 0.06$	$0.33^{abA} \pm 0.02$	$10.15^{bB} \pm 1.16$	$7.85^{bB} \pm 0.42$	$0.13^{aA} \pm 0.02$
FOCE50%	$27.43^{bC} \pm 2.48$	$0.85^{aA} \pm 0.06$	$0.33^{abA} \pm 0.05$	$10.08^{bA} \pm 1.74$	$8.25^{bA} \pm 0.13$	$0.15^{aA} \pm 0.01$
FOCE75%	$27.50^{bB} \pm 2.87$	$0.88^{aA} \pm 0.03$	$0.36^{bA} \pm 0.02$	$10.04^{bB} \pm 0.86$	$8.80^{bcB} \pm 0.80$	$0.15^{aA} \pm 0.02$
FOCE100%	$30.14^{bC} \pm 1.53$	$0.90^{aA} \pm 0.02$	$0.36^{bA} \pm 0.01$	$10.91^{bB} \pm 0.75$	$9.53^{cA} \pm 0.39$	$0.15^{aA} \pm 0.01$

$^{a-c}$ different lowercase letters indicate significant differences between GFBs with different FOCE levels at the same storage time ($p < 0.05$); $^{A-C}$ different capital letters indicate significant differences between storage times for the same bread ($p < 0.05$).
